# Prenatal and early life influences on epigenetic age in children: a study of mother–offspring pairs from two cohort studies

**DOI:** 10.1093/hmg/ddv456

**Published:** 2015-11-05

**Authors:** Andrew J. Simpkin, Gibran Hemani, Matthew Suderman, Tom R. Gaunt, Oliver Lyttleton, Wendy L. Mcardle, Susan M. Ring, Gemma C. Sharp, Kate Tilling, Steve Horvath, Sonja Kunze, Annette Peters, Melanie Waldenberger, Cavin Ward-Caviness, Ellen A. Nohr, Thorkild I. A. Sørensen, Caroline L. Relton, George Davey Smith

**Affiliations:** 1 MRC Integrative Epidemiology Unit, School of Social and Community Medicine,; 2 School of Social and Community Medicine, University of Bristol, Bristol BS8 2BN, UK,; 3 Human Genetics, David Geffen School of Medicine,; 4 Biostatistics, School of Public Health, University of California Los Angeles, Los Angeles, CA 90095, USA,; 5 Research Unit of Molecular Epidemiology, Helmholtz Zentrum München,; 6 Institute of Epidemiology II, Helmholtz Zentrum München, German Research Center for Environmental Health, Neuherberg, Germany,; 7 Research Unit for Gynaecology and Obstetrics, Institute of Clinical Research, University of Southern Denmark, Odense, Denmark,; 8 The Novo Nordisk Foundation Center for Basic Metabolic Research, Section on Metabolic Genetics, Faculty of Health and Medical Sciences, University of Copenhagen, Copenhagen, Denmark,; 9 Institute of Preventive Medicine, Bispebjerg and Frederiksberg Hospital, The Capital Region, Copenhagen, Denmark and; 10 Institute of Genetic Medicine, NewcastleUniversity, Newcastle upon Tyne NE1 3BZ, UK

## Abstract

DNA methylation-based biomarkers of aging are highly correlated with actual age. Departures of methylation-estimated age from actual age can be used to define epigenetic measures of child development or age acceleration (AA) in adults. Very little is known about genetic or environmental determinants of these epigenetic measures of aging. We obtained DNA methylation profiles using Infinium HumanMethylation450 BeadChips across five time-points in 1018 mother–child pairs from the Avon Longitudinal Study of Parents and Children. Using the Horvath age estimation method, we calculated epigenetic age for these samples. AA was defined as the residuals from regressing epigenetic age on actual age. AA was tested for associations with cross-sectional clinical variables in children. We identified associations between AA and sex, birth weight, birth by caesarean section and several maternal characteristics in pregnancy, namely smoking, weight, BMI, selenium and cholesterol level. Offspring of non-drinkers had higher AA on average but this difference appeared to resolve during childhood. The associations between sex, birth weight and AA found in ARIES were replicated in an independent cohort (GOYA). In children, epigenetic AA measures are associated with several clinically relevant variables, and early life exposures appear to be associated with changes in AA during adolescence. Further research into epigenetic aging, including the use of causal inference methods, is required to better our understanding of aging.

## Introduction

There has been considerable recent interest in epigenetic biomarkers of aging (1–6), which use an individual's DNA methylation data to estimate their ‘epigenetic age’, a concept that could be considered a form of biological age. The Horvath age estimation method (1) had a correlation of 0.96 between actual and epigenetic age, with individual estimates of epigenetic age within 3 years of actual age, on average. Epigenetic age has the potential to assess our biological age, but little is known about its relationship with our basic physiology. Moreover, several recent papers have found that the difference between epigenetic and actual age (known as age acceleration, denoted AA) has biological significance. A positive AA indicates an individual is ahead of their epigenetic clock, a negative AA (i.e. age deceleration) suggests an individual is behind their epigenetic clock. For example, AA has been found to be associated with obesity (7), Down's syndrome (8), HIV (9), physical and cognitive fitness (10) and all-cause mortality in older individuals (11). Furthermore, the epigenetic clock method has been applied to study aging in blood samples from subjects with a severe developmental disorder (12) and to estimate the ages of tissues from centenarians (13). However, there has been no investigation on prenatal and antenatal factors that affect AA in children. It is possible that the detrimental consequences of a higher AA may accrue over time, initiating in childhood. Conversely, it could be postulated that having a positive AA during early life and childhood is developmentally advantageous. To reflect this, we could refer to AA as an epigenetic measure of development in children, though for the sake of clarity, we will use AA throughout this article.

The Horvath age estimation method was developed using a training dataset of roughly 4000 samples from 20 healthy tissues and cell types (1). These datasets were assembled using a combination of Illumina HumanMethylation27 BeadChip (27K) and Illumina HumanMethylation450 BeadChip (450K) technology. The Horvath method uses 353 CpG sites which are common to both the 27 and 450K platforms to estimate age. A noteworthy advantage of this age estimation method is its applicability to most human tissues, cell types, and fluids (including blood). Freely available software that implements the method can be found online (http://labs.genetics.ucla.edu/horvath/htdocs/dnamage) (1). The Hannum age estimation method (2) was developed based on a single dataset (*n* = 656) of Illumina 450K methylation values taken from adult whole blood. It uses 71 CpG sites to estimate age. Although both of these age estimation methods have an unprecedented accuracy in whole blood, relatively little is known about exposures that affect epigenetic age.

The Accessible Resource for Integrated Epigenomic Studies (ARIES) (14) project has generated Illumina Infinium 450K array data from peripheral blood collected from 1018 women during pregnancy (antenatal) and again 17 years later (follow-up). Epigenetic data were generated from 1018 children of the aforementioned women from cord blood and peripheral blood drawn during both childhood (age 7) and adolescence (ages 15–17). We applied both the Horvath and Hannum methods to estimate the epigenetic age for each of these five time-points in ARIES.

In this article, we provide a comprehensive investigation of AA in children using data from three stages of early life (birth, 7 and 17). We also use linear mixed models to assess the associations between pre-birth exposures with average AA and changes in AA during childhood. We perform genetic analysis of epigenetic age, involving the application of a whole-genome restricted maximum likelihood (GREML) analysis for estimating the proportion of variation in AA that is attributable to all common genetic variants (referred to as SNP heritability).

## Results

There were 1018 different families in the ARIES sample, though only 914 (cord), 973 (childhood), 974 (adolescence), 951 (antenatal) and 970 (follow-up) had required methylation data available at their respective clinic to estimate epigenetic age. Sample characteristics are provided in Table 1. The GOYA study was used for replication of findings and includes DNA methylation from 981 newborns, along with clinical information on both mothers and children.

**Table 1.** Characteristics of the 1018 ARIES sample (continuous)

Group	Variable	Clinic		Mean/*N*	SD	Min	Max
*Children*	Age (years)	Childhood		7.49	0.15	7.10	9.08
		Adolescence		17.14	1.01	14.69	19.33
	Epigenetic age (years)	Birth		0.26	0.63	−0.59	16.68
		Childhood		8.25	2.42	2.50	24.80
		Adolescence		17.20	4.34	3.77	31.65
	Birth weight (g)	Birth		3488.2	487.29	1485.00	5140.00
	Gestational age (weeks)	Birth		39.56	1.52	30.00	44.00
	Height (cm)	Childhood		126.02	5.20	109.20	147.30
		Adolescence		172.37	9.15	146.70	203.00
	BMI (kg/m^2^)	Childhood		16.21	2.04	12.65	29.15
		Adolescence		22.61	3.90	14.74	50.06
	Sex	Birth	Boys	495 (48%)			
			Girls	527 (52%)			
*Mothers*	Age (years)	Antenatal		29.16	4.39	16.00	42.00
		Follow-up		47.38	4.45	34.52	60.00
	Epigenetic age (years)	Antenatal		30.17	6.72	0.33	53.77
		Follow-up		44.66	6.86	4.47	68.08
	Weight (kg)	Antenatal		61.64	10.51	40.00	122.27
	Height (cm)	Antenatal		164.56	6.59	144.78	185.42
	BMI (kg/m^2^)	Antenatal		22.77	3.66	14.23	45.24
		Follow-up		26.58	5.33	17.40	54.03
	Smoking	Antenatal	No	882 (88%)			
			Yes	117 (12%)			
	Alcohol	Antenatal	No	432 (44%)			
			Yes	570 (56%)			
	Breast fed	Antenatal	No	150 (15%)			
			Yes	828 (85%)			
	Education	Antenatal	CSE	88 (9%)			
			Voc.	72 (7%)			
			O Level	342 (34%)			
			A level	294 (30%)			
			Degree	205 (20%)			

BMI, body mass index; CSE, Certificate of Secondary Education; Voc., vocational.

### Replication of the accuracy of the epigenetic clock

**Figure 1.**
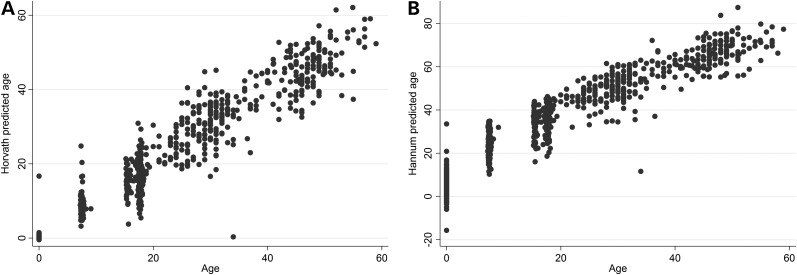
Horvath (left) and Hannum (right) predicted ages against actual ages from a random sample of 1018 individuals from the ARIES study.

**Figure 2.**
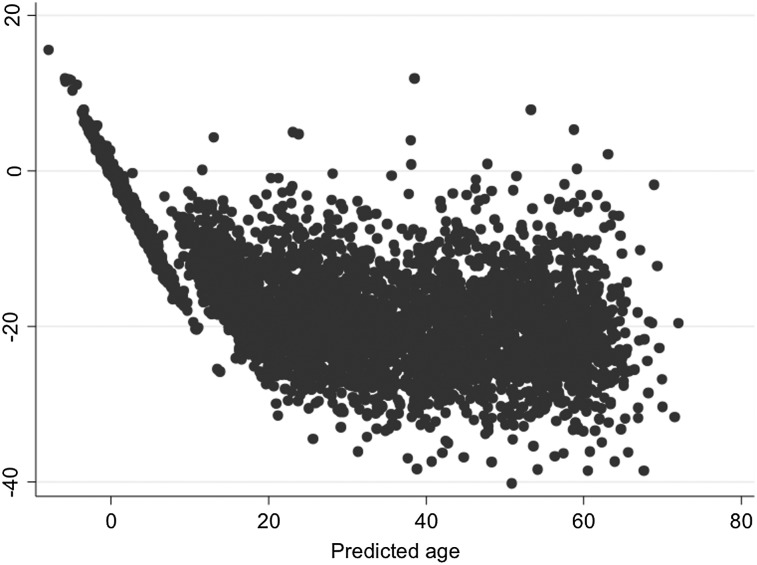
Bland–Altman plot of Horvath predicted age minus Hannum predicted age.

Taking a random sample of 1018 children and mothers from ARIES, there were correlations of 0.97 (Horvath) and 0.94 (Hannum) between epigenetic age and actual age, with an average absolute difference of 2.8 years (Horvath) and 16.8 years (Hannum) between epigenetic and actual ages. Apart from the mean absolute difference for the Hannum derived age estimate, our results replicate the original findings (Table 2, Fig. 1). The Bland–Altman plot in Figure 2 shows the disparity between the Horvath and Hannum methods, particularly in children, this is likely due to the fact that the Hannum method was developed using data from adults only. Furthermore, the correlation between epigenetic age estimated at birth and gestational age at delivery was small (Horvath *r* = 0.0033, Hannum *r* = 0.064).

**Table 2.** Performance of Horvath and Hannum predicted ages in the ARIES sample

Correlation between predicted and actual age	Pearson correlation coefficient			
Horvath	0.97			
Hannum	0.94			
Difference between actual and predicted age (years)	Mean	SD	Min	Max
Horvath	2.79	3.07	0.01	33.67
Hannum	16.75	7.26	0.09	36.41
Regression of predicted age on actual age	Coefficient	s.e.	*R*^2^	
Horvath	0.96	0.01	0.93	
Hannum	1.22	0.01	0.89

### Cross-sectional analysis

**Figure 3.**
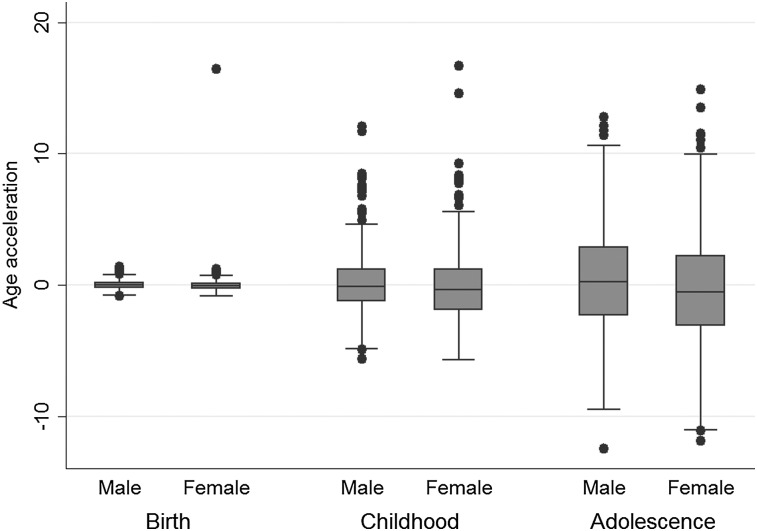
AA differences by sex over the life course.

**Figure 4.**
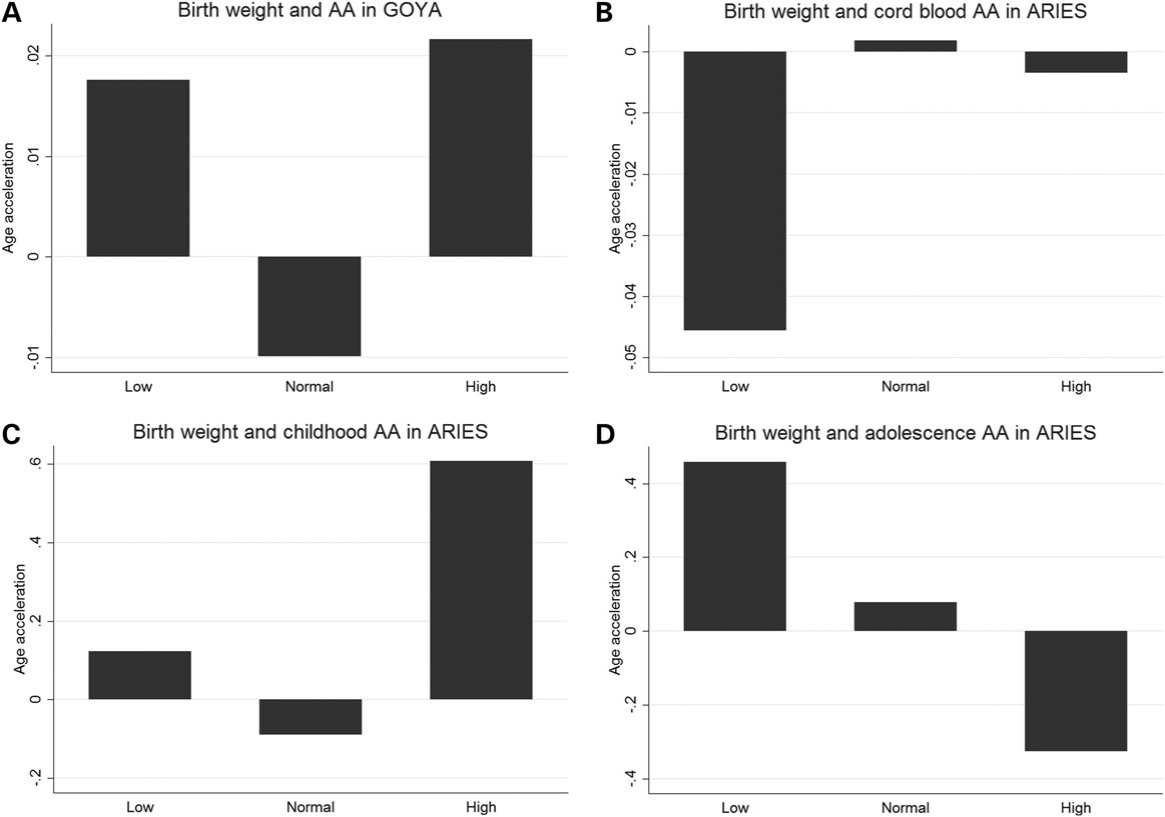
Birth weight category and AA in GOYA newborns (top left), ARIES newborns (top right), ARIES children (bottom left) and ARIES adolescents (bottom right).

When considering the relationship between epigenetic age and phenotypic traits in the ARIES children (Table 3), the strongest association was between maternal levels of selenium during pregnancy and offspring AA at age 7 (*r* = −0.140; *P* = 0.009) and at birth (*r* = −0.103, *P* = 0.061). Girls tended to have lower AA on average than boys, and this association strengthened over the life course (Fig. 3) from age 7 (*r* = −0.055, *P* = 0.085) to age 17 (*r* = 0.082, *P* = 0.01). Birth weight in kilograms was positively associated with AA at age 7 (*r* = 0.079, *P* = 0.014) but negatively associated by age 17 (*r* = −0.068, *P* = 0.035); birth weight broken into low, normal and high categories (Fig. 4) was associated with AA at age 7 (*F* = 5.13, *P* = 0.006), but not at birth (*F* = 0.06, *P* = 0.941) or age 17 (*F* = 0.70, *P* = 0.496). Positive associations were identified between offspring AA and systolic BP (*r* = 0.093, *P* = 0.009), maternal weight (*r* = 0.074, *P* = 0.026), maternal BMI (*r* = 0.073, *P* = 0.029), maternal smoking (*r* = 0.097, *P* = 0.004) and having been delivered following a caesarean section (*r* = 0.067, *P* = 0.048). A negative association was found between offspring AA and maternal cholesterol (*r* = −0.103, *P* = 0.031).

**Table 3.** Correlation analysis and ANOVA of AA for ARIES children

Clinical variables^a^	Clinic for AA	Correlation (or *F*-statistic*)	*P*-value
Sex (*M* = 0, *F* = 1)	Birth	−0.019	0.565
	Childhood	−0.055	0.085
	Adolescence	−0.082	0.010
Parity	Birth	−0.018	0.596
	Childhood	0.017	0.600
	Adolescence	−0.071	0.030
Caesarean (*N* = 0, *Y* = 1)	Birth	0.067	0.048
	Childhood	0.006	0.846
	Adolescence	0.019	0.555
Birth weight (kg)	Birth	0.011	0.734
	Childhood	0.079	0.014
	Adolescence	−0.068	0.035
Birth weight (category)^b^	Birth	0.01	0.988
	Childhood	2.40	0.092
	Adolescence	3.73	0.024
Gestational age at delivery (week)	Birth	0.003	0.922
	Childhood	0.029	0.363
	adolescence	−0.048	0.134
Breast feeding (*N* = 0, *Y* = 1)	Birth	0.035	0.301
	Childhood	−0.010	0.756
	Adolescence	0.026	0.434
Maternal alcohol (*N* = 0, *Y* = 1)	Birth	0.034	0.307
	Childhood	−0.011	0.732
	Adolescence	−0.015	0.647
Maternal smoking (*N* = 0, *Y* = 1)	Birth	0.097	0.004
	Childhood	0.021	0.518
	adolescence	−0.016	0.616
Maternal education^b,c^	Birth	0.55	0.699
	Childhood	0.37	0.831
	Adolescence	1.40	0.232
Maternal weight (kg)	Birth	0.002	0.948
	Childhood	0.074	0.026
	Adolescence	0.006	0.855
Maternal height (cm)	Birth	0.004	0.907
	Childhood	−0.010	0.769
	Adolescence	−0.012	0.719
Maternal BMI (kg/m^2^)	Birth	0.003	0.925
	Childhood	0.073	0.029
	Adolescence	0.005	0.877
Maternal cholesterol (mmol/l)	Birth	−0.043	0.384
	Childhood	−0.103	0.031
	Adolescence	0.016	0.737
Maternal cadmium (µg/l)	Birth	−0.120	0.067
	Childhood	0.034	0.592
	Adolescence	0.051	0.418
Maternal lead (µg/l)	Birth	−0.037	0.499
	Childhood	−0.002	0.971
	Adolescence	−0.072	0.180
Maternal selenium (µg/l)	Birth	−0.103	0.060
	Childhood	−0.137	0.009
	Adolescence	0.011	0.837
Maternal mercury (µg/l)	Birth	−0.030	0.601
	Childhood	−0.023	0.673
	Adolescence	−0.006	0.920
Maternal vitamin D (nmol/l)	Birth	−0.052	0.200
	Childhood	−0.002	0.950
	Adolescence	−0.009	0.823
Maternal cotinine (ng/ml)	Birth	−0.022	0.675
	Childhood	0.059	0.238
	Adolescence	−0.019	0.707
Maternal age (years)	Birth	−0.027	0.427
	Childhood	0.028	0.388
	Adolescence	−0.045	0.165
Height (cm; measured at 7)^d^	Childhood	0.061	0.058
	Adolescence	0.058	0.073
BMI (kg/m^2^; measured at 7)	Childhood	0.037	0.249
	Adolescence	0.005	0.880
Cotinine (ng/ml; measured at 7)	Childhood	0.071	0.031
	Adolescence	0.033	0.313
Leptin (ng/ml; measured at 9)	Adolescence	0.024	0.523
Interleukin 6 (pg/ml; measured at 9)	Adolescence	0.068	0.065
Height (cm; measured at 17)	Adolescence	0.051	0.144
BMI (kg/m^2^; measured at 17)	Adolescence	0.017	0.620
Total cholesterol (mmol/l; measured at 17)	Adolescence	−0.030	0.433
HDL cholesterol (mmol/l; measured at 17)	Adolescence	0.030	0.425
Triglycerides (mmol/l; measured at 17)	Adolescence	−0.010	0.794
LDL cholesterol (mmol/l; measured at 17)	Adolescence	−0.014	0.717
C-reactive protein (mg/l; measured at 15)	Adolescence	0.004	0.905
Systolic BP (mmHg; measured at 17)	Adolescence	0.093	0.009
Diastolic BP (mmHg; measured at 17)	Adolescence	−0.055	0.122

^a^Measured at birth/in pregnancy unless otherwise stated.

^b^One-way ANOVA *F*-statistic and associated *P*-value reported for ordinal variables.

^c^Maternal education is ordinal: CSE, vocational, O level, A level, degree.

^d^Association analysis only performed against AA measured at same time or before clinical variable.

### Longitudinal analysis

Correlation of epigenetic age between ARIES time-points became stronger as age increased; birth and childhood samples had the lowest correlation (*r* = 0.109, *P* = 0.0012), followed by birth and adolescent samples (*r* = 0.174, *P* < 0.0005), while the strongest correlation in the offspring was between childhood and adolescent samples (*r* = 0.260, *P* < 0.0005). Correlation was even stronger in the ARIES mother's epigenetic age between the antenatal and follow-up samples (*r* = 0.498, *P* < 0.0005).

In the longitudinal analysis of AA in the ARIES children (Table 4), maternal alcohol consumption during pregnancy had a negative association with average AA during childhood and adolescence (children of drinkers had 1.3 years lower AA on average, 95% CI −0.11, 2.7). However, this difference was resolved over time, with the offspring of drinkers having increased growth of AA during childhood and adolescence (0.19 years of AA per year of life, 95% CI 0.064, 0.308). There were other associations between average AA in offspring and maternal weight, height, BMI, cadmium and cotinine. There was a positive association between maternal smoking and changes in AA during childhood, with children of smokers having increased growth of AA (0.22 years of AA per year of life, 95% CI 0.07, 0.36). There were other associations between AA changes and sex, parity and maternal cadmium.

**Table 4.** Longitudinal analysis of AA

Clinical variable	Association with average AA (years) during childhood and adolescence	95% CI	*P*-value	Associations with changes in AA (AA years per year of life) during childhood and adolescence	95% CI	*P*-value
Sex (*M* = 0,*F* = 1)	−0.499	(−1.866,0.867)	0.47	−0.136	(−0.251, −0.02)	0.022
Caesarean (*N* = 0, *Y* = 1)	−1.67	(−4.61,1.27)	0.27	0.201	(−0.03,0.433)	0.088
Parity	0.179	(−0.809,1.167)	0.72	−0.038	(−0.11,0.035)	0.308
Birth weight (kg)	0.0003	(−0.001,0.002)	0.72	−0.00004	(−0.00017,0.00009)	0.583
Gestational age (weeks)	−0.027	(−0.674,0.621)	0.94	0.007	(−0.038,0.053)	0.752
Maternal age (years)	−0.003	(−0.198,0.193)	0.98	0.001	(−0.013,0.015)	0.871
Maternal weight (kg)	−0.892	(−1.686, −0.097)	0.03	0.004	(−0.002,0.01)	0.178
Maternal height (cm)	0.64	(0.038,1.242)	0.04	0.003	(−0.004,0.011)	0.366
Maternal BMI (kg/m^2^)	2.212	(0.036,4.387)	0.05	0.011	(−0.009,0.03)	0.285
Maternal smoking (*N* = 0, *Y* = 1)	1.822	(−1.271,4.915)	0.25	0.216	(0.073,0.36)	0.003
Maternal alcohol (*N* = 0, *Y* = 1)	−1.297	(−2.705,0.11)	0.07	0.186	(0.064,0.308)	0.003
Maternal cholesterol (mmol/l)	0.257	(−0.301,0.815)	0.37	−0.016	(−0.06,0.028)	0.467
Maternal cadmium (µg/l)	−1.616	(−3.25,0.018)	0.05	0.092	(0.014,0.171)	0.021
Maternal lead (µg/l)	−0.078	(−0.578,0.421)	0.76	−0.02	(−0.061,0.021)	0.345
Maternal selenium (µg/l)	0.00027	(−0.021,0.022)	0.98	−0.001	(−0.003,0.001)	0.194
Maternal mercury (µg/l)	0.42	(−0.375,1.214)	0.30	−0.01	(−0.071,0.051)	0.749
Maternal vitamin D (nmol/l)	−0.002	(−0.025,0.02)	0.83	0.00038	(−0.002,0.002)	0.714
Maternal cotinine (ng/ml)	−0.001	(−0.001,0.000004)	0.05	0.00002	(−0.00002,0.00005)	0.408
Maternal education						
CSE	Reference		0.76	Reference		0.183
Voc.	−0.304	(−1.841,1.234)		0.11	(−0.019,0.239)	
O Level	−0.238	(−1.556,1.081)		0.038	(−0.073,0.148)	
A Level	−0.38	(−1.694,0.933)		0.058	(−0.052,0.167)	
Degree	−0.507	(−1.865,0.85)		0.103	(−0.01,0.215)	

BMI, body mass index; CSE, Certificate of Secondary Education; Voc., vocational.

#### Replication in the GOYA study

Newborn girls had a 0.06 year lower average AA than boys in the GOYA study (95% CI 0.03, 0.09 years, *P* < 0.0005), which replicates our own finding in children and adolescents in ARIES. Birth weight in kilograms was positively associated with newborn AA in GOYA (0.04 years per kilogram of birth weight, 95% CI 0.02, 0.07 years, *P* = 0.002); however, there was little evidence that birth weight broken into categories of low, normal and high was associated with newborn AA (Fig. 4). In the cross-sectional GOYA analysis, there was little evidence for associations between maternal alcohol consumption (offspring of drinkers had 0.011 years higher AA on average, 95% CI −0.03, 0.05 years, *P* = 0.589) or maternal smoking (offspring of smokers had 0.02 years lower AA on average, 95% CI −0.01, 0.04 years lower, *P* = 0.206) during pregnancy and offspring AA at birth.

### Whole-genome GREML analysis

**Figure 5.**
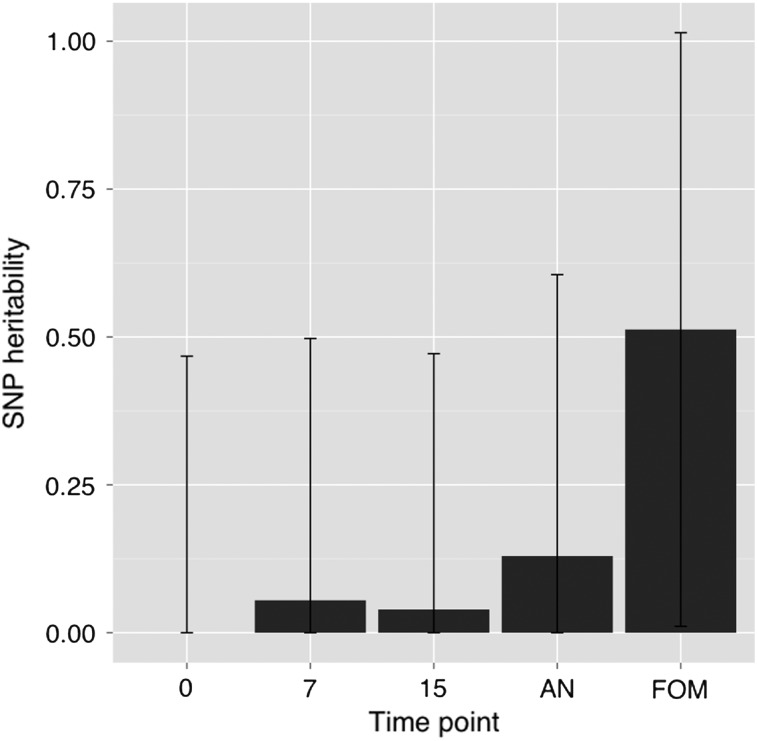
SNP heritability estimates for AA at each time point using unrelated individuals. There is a larger genetic component when individuals increase in age.

**Figure 6.**
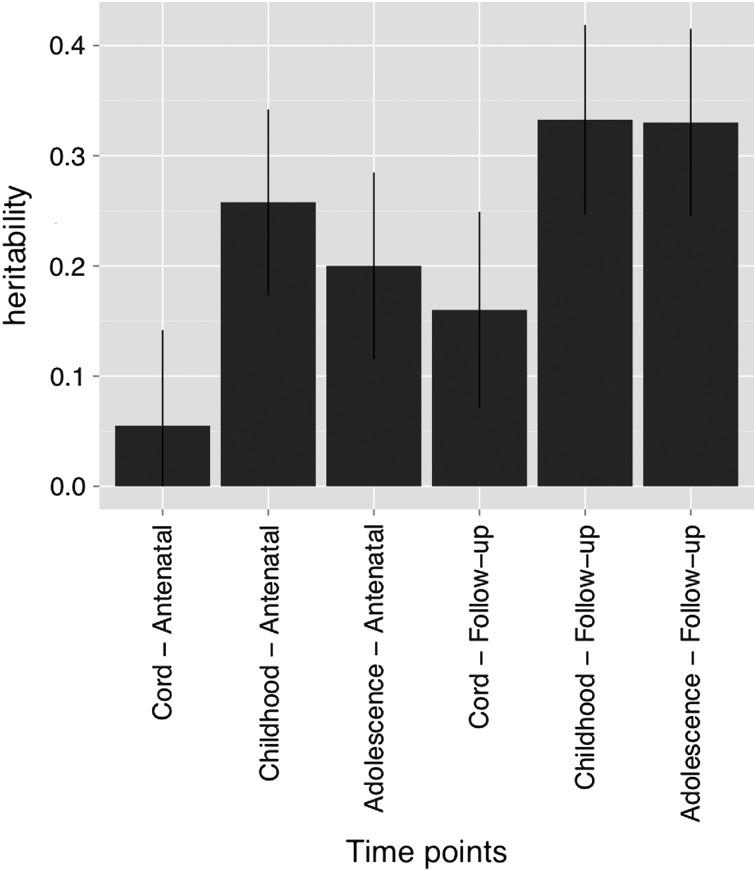
Heritability analysis of AA. The analysis was performed for each mother–child time-point combination (this was done to improve power), showing a general increase in genetic variance for AA as individuals get older. AN, AnteNatal clinic; FOM, Follow-on Mother's clinic.

Standard errors were very large due to small sample size, but there was a trend of increasing SNP heritability point estimates for AA as participant age increases (Fig. 5). Combining mothers and children to improve precision yields a similar pattern of increasing point estimates with increasing age, with the oldest age combination (15-year-old children and middle aged mothers) giving an SNP heritability estimate of 0.37 (s.e. 0.25, *P* = 6.6 × 10^−5^) for the familial component of AA (Fig. 6).

## Discussion

### Summary

Using repeated blood draws from a cohort of 1018 mothers and children in the UK, we have provided a thorough investigation of epigenetic age in children. We found associations between AA and sex, birth weight, caesarean section delivery and several maternal characteristics, namely smoking in pregnancy, weight, BMI, selenium and cholesterol level. In further longitudinal analysis of the ARIES offspring, we observed an association between maternal alcohol consumption and smoking during pregnancy with changes in AA during development. Offspring of non-drinkers had higher AA on average at birth, but this appeared to resolve during childhood. Offspring of smokers had higher AA on average and this difference became larger during childhood and adolescence. This finding is congruent with a recent longitudinal study of DNA methylation levels in 28 smoking-related CpG sites in the ARIES children (15). Differences identified at birth in offspring tended to persist at maternal smoking-related CpG sites. Results on birth weight (16) and maternal BMI (17)-related CpG sites are more consistent with the maternal alcohol finding, i.e. a resolution of birth differences during early life. Using an independent cohort (the GOYA sub-study of the Danish National Birth Cohort), we found similar associations between sex, birth weight and AA in children.

The accuracy of the Horvath method suggests a wide range of potential uses, including forensic applications which have begun to be explored (6,18). In this paper, we have begun to investigate how and why individuals differ from their epigenetic age, with findings in the ARIES cohort related to BMI in adults. While there was no clear replication of this BMI-AA association in an independent cohort (the KORA cohort), the modelling of AA is in its infancy, and studies have found this BMI-AA association in liver tissue (7). The lack of correlation between AA and several clinical variables may also indicate that AA reflects an ‘intrinsic’ aging rate that is independent of various aging factors. The observation that the estimated genetic component of AA increased in older study participants may indicate that the AA measure is more biologically meaningful in adults rather than children, though alternatively it could be a reflection of a decreasing environmental influence on DNA methylation patterns over time. This accords with our finding of strengthening within subject correlation over time, which suggests the period of rapid early life changes in methylation affects epigenetic age during development to a greater extent than adulthood changes in methylation. Future research is needed to understand why the correlation of epigenetic age increases as age increases. This may be due to the rapid change of methylation in children compared with adults. Indeed, the epigenetic clock has been found to be more accurate in children, the younger the subjects, the more accurate the epigenetic age (1,12).

Our heritability estimates suffered from a lack of power, but were consistent with previous findings (11). The heritability estimate from our study (*h* = 0.37) is lower than that reported Horvath (1) (*h* = 1.0), which was based on a small number of cord blood samples from twin pairs. Both of these heritability estimates were based on relatively few samples. Future large scale studies will be needed to arrive at precise estimates of the heritability of AA in newborns and minors. While our heritability estimate may seem low, empirical evidence (19) has suggested that fitness related traits tend to have lower heritability than morphological traits because selection acts to purify deleterious genetic variation, and one might consider age accelerated residuals in the former category.

### Strengths

The ARIES data include five time-points at which DNA methylation was measured in the same families and is a unique epigenetic resource. This allowed us to replicate the Horvath epigenetic clock finding in a longitudinal cohort. Furthermore, ARIES is a subsample within Avon Longitudinal Study of Parents and Children (ALSPAC), which has measured thousands of variables over many years. Thus, we were able to investigate the relationship between AA and a wide range of clinically relevant factors, as well as model changes in AA over time. Epigenetic age showed substantial inter-individual correlation over time, with a stronger correlation in the mothers than in their offspring. Among the children, the inter-individual correlation strengthened with actual age. We replicated our finding that boys and those with larger birth weight have higher AA in childhood in an independent study (GOYA).

### Limitations

Our findings are limited to DNA extracted from peripheral blood (or cord blood in infants) so future research should evaluate whether these variables relate to AA measures in other tissues or cell types. For example, Horvath *et al*. (7) showed that BMI is strongly correlated with AA in liver, but much less so in other tissues such as adipose, muscle, or blood. Although the 1018 mother–child pairs are an excellent resource, we still have low power to run heritability (GREML) analyses. These approaches generally need several thousand samples in order to account for multiple testing. AAs were adjusted for estimated cell-type proportions; however, since these were estimated from the DNA methylation data rather than actually measured, it is unclear whether incomplete adjustment has affected our findings. Indeed, Marioni (11) finds that the AA measure estimated by the Hannum method is significantly correlated with several measures of cell counts (e.g. the abundance of senescent CD8 T cells and naive CD8 T cells). The Horvath-based AA measure is less confounded by the relative abundance of blood cell types, which probably reflects that it was formulated from multiple different tissues and cell types. We were unable to replicate our findings on maternal smoking or alcohol consumption on offspring AA, perhaps due to the fact that the replication cohort was cross-sectional, as opposed to our longitudinal design. Assessing the causal relationship between exposures and AA (through Mendelian randomization, e.g. 20,21) is underpowered in our current data. For example, we have an estimated 6% power to detect a causal link between maternal smoking and offspring AA (see Supplementary Material).

### Conclusions

Using serially collected DNA methylation, we have provided evidence that epigenetic AA measures are associated with several clinically relevant variables in both mothers and children. We have found and replicated strong associations between prenatal and early life factors and epigenetic age in children. Further research [including the use of causal inference methods such as Mendelian randomization (20,21)] and replication are needed to understand the key drivers behind our epigenetic clock and its relation to development and aging.

## Materials and Methods

### Study population

This study used DNA methylation data generated under the auspices of the ALSPAC (22,23). ALSPAC recruited 14 541 pregnant women with expected delivery dates between April 1991 and December 1992. Of these initial pregnancies, there were 14 062 live births and 13 988 children who were alive at 1 year of age. The study website contains details of all the data that are available through a fully searchable data dictionary (http://www.bris.ac.uk/alspac/researchers/data-access/data-dictionary).

As part of the ARIES (14) project (http://www.ariesepigenomics.org.uk), a subsample of 1018 ALSPAC child–mother pairs had DNA methylation measured using the Infinium HumanMethylation450 BeadChip (Illumina, Inc.) (24). In this study, we use DNA methylation data generated from cord blood and peripheral blood samples at age 7 and again at age 15 or 17 years, leading to three measurements of DNA methylation per child. DNA methylation data were also generated on the mothers of these children, from blood samples taken during pregnancy and at a follow-up clinic 17 years later using the same platform.

### Laboratory methods, quality control and pre-processing

All DNA methylation wet-lab and pre-processing analyses were performed at the University of Bristol as part of the ARIES project. Following extraction, DNA was bisulfite converted using the Zymo EZ DNA MethylationTM kit (Zymo, Irvine, CA, USA). Infinium HumanMethylation450 BeadChips were used to measure genome-wide DNA methylation levels at over 485 000 CpG sites. The arrays were scanned using an Illumina iScan, with initial quality review using GenomeStudio. The level of methylation is expressed as a ‘beta’ value (*β*-value), ranging from 0 (no cytosine methylation) to 1 (complete cytosine methylation). β-Values are reported as percentages. Several quality control steps were included in the laboratory pipeline which are described in detail elsewhere (16).

### Epigenetic age

Using the online epigenetic clock calculator (http://labs.genetics.ucla.edu/horvath/dnamage/), we obtained DNA methylation predicted age using both the Horvath (1) and Hannum (2) methods, using raw β-values from the ARIES study. The Horvath method is based on 353 CpG sites, while the Hannum prediction uses 71 sites. Along with predicted age, the online calculator estimates cell-type proportions and calculates raw AA differences (predicted-actual age) and AA residuals (the residuals from a linear regression of epigenetic age on actual age, which we call age acceleration and denote AA). AA is uncorrelated with actual age and contains information about the methylation age profiles of each sample, i.e. a positive residual corresponds to an individual whose methylation age is ahead of their actual age and vice versa. The online calculator also provides AA adjusted for cell heterogeneity, by way of estimated cell-type proportions.

### Replication of previous findings

Using the ARIES data, we sought to replicate previous findings which found a correlation of 0.96 [both Horvath (1) and Hannum (2)] and an average difference of 2.9 years [Horvath (1)) or 3.9 years (Hannum (2)] between predicted and actual age. To coincide with the spread of ages in the original work, we randomly selected a sample of 1018 different individuals from across the five time-points in ARIES and compared actual against DNA methylation predicted age from each method.

### Variables assessed

To investigate associations with AA, we assessed biological measures from the ARIES children and their mothers. For the children, these were sex (coded boy 0, girl 1), birth weight [both continuous and broken into low (<2.5 kg), normal and high (>4 kg) categories to account for U-shaped trend between birth weight and chronic disease risk (25)], gestational age at delivery, parity and delivery method (natural/caesarean). We also tested whether maternal characteristics during pregnancy were related to offspring AA, including maternal age, height, weight, BMI, alcohol consumption (Y/N), smoking status (Y/N) and highest education level achieved; we also investigated offspring AA against maternal levels of cholesterol, cadmium, lead, mercury, selenium, cotinine and vitamin D during pregnancy. These represent 19 variables which could be associated with AA from birth. We supplement these with further variables which could be associated with AA at either the childhood (age 7) or adolescent (ages 15–17) clinics: whether the child was breast fed (Y/N), cotinine (at 7), leptin (at 9), interleukin-6 (at 9), C-reactive protein (at 15), cholesterol (total, LDL, HDL, triglycerides at 17) and diastolic/systolic blood pressure (at 17).

For the ARIES mothers (results provided in Supplementary Material), there were two time-points with DNA methylation and clinical variables available. During pregnancy, we compared AA against contemporaneous measures of height, BMI, smoking, alcohol intake, education, socio-economic position (occupation) and total cholesterol. At the mother's follow-up clinic, we included height, BMI, smoking, alcohol intake, education, socio-economic position (occupation); systolic and diastolic blood pressure; total, HDL and LDL cholesterol, triglyceride and C-reactive protein levels.

### Statistical analysis

All AAs were adjusted for estimated cell-type composition using the online calculator. Pearson's correlation, *R*^2^ and mean difference between predicted and actual age were used to assess the performance of the age prediction methods. A Bland–Altman plot was used to investigate the agreement of age predictions from each method.

Pearson correlation tests were used to investigate the linear relationship between Horvath AA and continuous or binary variables, one-way analysis of variance (ANOVA) was used to compare Horvath AA across categories of maternal education and birth weight category. In total, there were 78 (children) and 33 (mother) tests such yielding a Bonferroni correction *P*-value threshold of 4.5 × 10^−4^ (0.05/111). We report the relationship between adult AA and clinical variables in Supplementary Material, as the focus of the paper is on AA in children.

Replication of findings in the ARIES children was carried out using cord blood samples from the GOYA study (sample size 981, mean maternal age at pregnancy 29.2, range 18–44) (26,27) which had phenotypic information available on mothers. The GOYA study is a subsample of the Danish National Birth Cohort, which took the most obese mothers from the DNBC along with controls who were randomly sampled from the remaining mothers in the DNBC. For analysis of the GOYA data, we present results of linear regression of AA on phenotypes adjusted for case–control status. Replication of findings in the ARIES mothers was attempted in the women of the KORA cohort (sample size 921, mean age 60.6, range 39–81, further details provided in Supplementary Material) (28).

Correlation between epigenetic ages at different clinics was used to investigate the strength of association within subjects over the life course. Mutually adjusted, linear mixed models (29) were then used in the offspring data to investigate the effect of the 19 early life exposures (described above) on average AA and also AA changes during childhood and adolescence. With 19 exposures, the Bonferroni corrected in this test *P*-value is 0.05/20 = 0.0263. This analysis was not repeated in the mothers as early life exposures were not present for them.

### Whole-genome methods

This analysis attempted to estimate SNP heritability of AA using GCTA software (30). For each time point ∼1.1 million Hapmap3 (31) SNPs (minor allele frequency > 0.01 and imputation info >0.8) were used to construct a genetic relationship matrix and SNP heritability (proportion of phenotypic variance explained by all SNPs) was estimated for the AA phenotype, fitting sex and 10 principle components as covariates. These tag SNPs were used to reduce bias in the SNP heritability estimation that can be incurred in the presence of very large levels linkage disequilibrium. Any pairs of individuals with relatedness >0.05 were excluded from the analysis. In order to improve power, we also attempted to estimate heritability by combining mothers and children together. A likelihood ratio test was used to obtain *P*-values using a *χ*^2^ test with 2 degrees of freedom. In order to estimate true heritability, the same procedure was followed except all individuals who were not mother–child pairs had genetic relatedness set to 0 in the genetic relationship matrix. This provides an upper bound the heritability estimate as it is liable to also include common environmental effects. Nevertheless, it is an orthogonal approach to the GREML analysis.

## Ethical Approval

Ethical approval for the study was obtained from the ALSPAC Ethics and Law Committee and the Local Research Ethics Committees.

## Supplementary Material

Supplementary Material is available at *HMG* online.

## Funding

This research was specifically funded by UK Economic & Social Research Council grant RES-060-23-0011, UK Medical Research Council grants G0601625, G0600705 and MR/L011824/1, and European Research Council grant 269874. ARIES was funded by the BBSRC (BBI025751/1 and BB/I025263/1). Core program support for ALSPAC is provided by the Medical Research Council (MRC) and the Wellcome Trust (Grant ref: 102215/2/13/1) and the University of Bristol. Supplementary funding to generate DNA methylation data which is (or will be) included in ARIES has been obtained from the MRC, ESRC, NIH and other sources. ARIES is maintained under the auspices of the MRC Integrative Epidemiology Unit at the University of Bristol (MC_UU_12013/2, MC_UU_12013/8, MRC_UU_12013/9). DNA methylation was obtained for the GOYA cohort using funding from the MRC IEU. The KORA cohort was supported by the German Federal Ministry of Education and Research (BMBF) within the framework of the e:Med research and funding concept (grant number 01ZX1313A-2014). Funding to pay the Open Access publication charges for this article was provided by the University of Bristol - RCUK Open Access fund.

## Supplementary Material

Supplementary Data
