# Supplementary material for: Prenatal and early life influences on epigenetic age in children: a study of mother–offspring pairs from two cohort studies
Source: Hum Mol Genet. 2015 Nov 5;25(1):191–201. doi: 10.1093/hmg/ddv456 (PMC4690495; doi:10.1093/hmg/ddv456)
Supplement: Supplementary Data [file supp_25_1_191__index.html]

Prenatal and early life influences on epigenetic age in children: a study of mother–offspring pairs from two cohort studies — Prenatal and early life influences on epigenetic age in children: a study of mother–offspring pairs from two cohort studies — Supplementary Data 

# Prenatal and early life influences on epigenetic age in children: a study of mother–offspring pairs from two cohort studies

## Supplementary Data

Supplementary Data

- Supplementary Data - Doc file
