# Supplementary material for: Prenatal and early life influences on epigenetic age in children: a study of mother–offspring pairs from two cohort studies
Source: Hum Mol Genet. 2015 Nov 5;25(1):191–201. doi: 10.1093/hmg/ddv456 (PMC4690495; doi:10.1093/hmg/ddv456)
Supplement: Supplementary Data [file supp_ddv456_ddv456supp.doc]

**Supplementary Material**

***Correlation analysis for ARIES mother’s epigenetic age***

There was strong evidence that BMI was positively associated with AA (r=0.12; p=0.00099; Table S1) at the later time point assessed in ARIES mothers (i.e. a follow-up clinic 17 years after delivery). We therefore attempted to replicate this finding in the KORA cohort, where no strong evidence for this association was found (r=0.04; p=0.25). C-reactive protein was also associated with AA in the ARIES mothers (r=0.096, p=0.008), and this association remained after adjusting for BMI (r=0.04, p=0.011). Positive associations were identified between adult AA and height (r=0.081, p=0.015), selenium (r=0.122, p=0.025) and triglycerides (r=0.081, p=0.025). Weak negative associations were observed between maternal AA and smoking (r=-0.071, p=0.03), alcohol consumption (-0.073, p=0.025) and HDL cholesterol level (r=-0.071, p=0.051).

***Power calculation for Mendelian randomisation***

We carried out a power calculation for using Mendelian randomisation to check for a causal link between maternal smoking and AA. The following inputs, required to calculate power, were taken from the observed associations in the ARIES data. These were n=894 families with maternal smoking information and AA in offspring available, an observed regression coefficient of 0.2 for the effect of maternal smoking on offpsring AA, an R-squared of 0.01 for the relationship between maternal smoking and offspring AA, variance of smoking being 0.1 in ARIES and variance of AA at birth being 0.6 in ARIES. Using these numbers, we estimate that these data have 6% power to detect a causal association.

Table S1: Correlation analysis of age acceleration for ARIES mothers

| **Clinical Variables[[1]](#footnote-2)** | **Clinic for AA** | **Correlation** | **p-value** |
| --- | --- | --- | --- |
| Alcohol (N=0, Y=1) | *Antenatal* | -0.073 | 0.025 |
|  | *Follow-up* | -0.050 | 0.133 |
| Smoking (N=0, Y=1) | *Antenatal* | -0.071 | 0.030 |
|  | *Follow-up* | -0.011 | 0.746 |
| Education[[2]](#footnote-3) | *Antenatal* | 0.017 | 0.607 |
|  | *Follow-up* | 0.047 | 0.165 |
| Weight (kg) | *Antenatal* | 0.037 | 0.260 |
|  | *Follow-up* | 0.018 | 0.601 |
| Height (cm) | *Antenatal* | 0.081 | 0.015 |
|  | *Follow-up* | 0.017 | 0.627 |
| BMI (kg/m^2^) | *Antenatal* | 0.059 | 0.078 |
|  | *Follow-up* | -0.002 | 0.948 |
| Total cholesterol (mmol/L) | *Antenatal* | 0.066 | 0.170 |
|  | *Follow-up* | 0.033 | 0.493 |
| Cadmium (µg/L) | *Antenatal* | -0.084 | 0.189 |
|  | *Follow-up* | -0.121 | 0.063 |
| Lead (µg/L) | *Antenatal* | -0.038 | 0.479 |
|  | *Follow-up* | 0.020 | 0.717 |
| Selenium (µg/L) | *Antenatal* | -0.065 | 0.224 |
|  | *Follow-up* | 0.122 | 0.025 |
| Mercury (µg/L) | *Antenatal* | 0.020 | 0.722 |
|  | *Follow-up* | 0.075 | 0.181 |
| Vitamin D (nmol/L) | *Antenatal* | 0.036 | 0.370 |
|  | *Follow-up* | -0.012 | 0.778 |
| Cotinine (ng/ml) | *Antenatal* | -0.027 | 0.594 |
|  | *Follow-up* | -0.027 | 0.603 |
| BMI (kg/m^2^; follow-up measured) | *Follow-up* | 0.115 | 0.0010 |
| Systolic BP (mmHg; follow-up measured) | *Follow-up* | -0.011 | 0.764 |
| Diastolic BP (mmHg; follow-up measured) | *Follow-up* | 0.026 | 0.454 |
| Total cholesterol (mmol/L; follow-up measured) | *Follow-up* | -0.050 | 0.169 |
| HDL cholesterol (mmol/L; follow-up measured) | *Follow-up* | -0.071 | 0.051 |
| Triglyceride (mmol/L; follow-up measured) | *Follow-up* | 0.081 | 0.025 |
| LDL cholesterol (mmol/L; follow-up measured) | *Follow-up* | -0.041 | 0.255 |
| C-reactive protein (mg/L; follow-up measured) | *Follow-up* | 0.096 | 0.008 |

1. measured during pregnancy unless otherwise stated [↑](#footnote-ref-2)
2. maternal education is ordinal: CSE, vocational, O level, A level, degree [↑](#footnote-ref-3)
